# *In vivo* canine study of three different coatings applied to p64 flow-diverter stents: initial biocompatibility study

**DOI:** 10.1186/s41747-018-0084-z

**Published:** 2019-01-22

**Authors:** Rosa Martínez Moreno, Pervinder Bhogal, Tim Lenz-Habijan, Catrin Bannewitz, Adnan Siddiqui, Pedro Lylyk, Ralf Hannes, Hermann Monstadt, Hans Henkes

**Affiliations:** 1 Hospital Universitario Virgen de las Nieves, Granada, Spain; 2 Department of Interventional Neuroradiology, The Royal London Hospital, Whitechapel Road, London, E1 1BB UK; 3 phenox GmbH, Bochum, Germany; 4 Jacobs School of Medicine and Biomedical Science, University of Buffalo, Buffalo, NY USA; 5 Clinica Sagrada Familia, ENERI, Buenos Aires, Argentina; 6 Neurozentrum, Klinikum Stuttgart, Stuttgart, Germany; 7 Medical Faculty, University Duisburg-Essen, Essen, Germany

**Keywords:** Biocompatible materials, Disease models (animal), Intracranial aneurysm, Materials testing, Stents

## Abstract

**Background:**

Flow-diverter stents (FDSs) have revolutionised the treatment of intracranial aneurysms. However, associated dual antiplatelet treatment is mandatory. We investigated the biocompatibility of three proprietary antithrombogenic coatings applied to FDSs.

**Methods:**

After Institutional Animal Care and Use Committee approval, four domestic juvenile female dogs (weight 19.9 ± 0.9 kg, mean ± standard deviation) were commenced on three different oral antiplatelet regimes: no medication (*n* = 1), acetylsalicylic acid (*n* = 2), and acetylsalicylic acid and clopidogrel (*n* = 1). Four p64 FDSs were randomly implanted into the subclavian, common carotid, and external carotid arteries of each dog, including both uncoated p64 stents and p64 stents coated with three different antithrombogenic hydrophilic coating (HPC). Angiography and histological examinations were performed. Wilcoxon/Kruskal-Wallis and ANOVA were used with *p* value < 0.05 considered as significant.

**Results:**

Minimal inflammatory cell infiltration and no device-associated granulomatous cell inflammation were observed. No significant difference in adventitial inflammation (*p* = 0.522) or neointimal/medial inflammation (*p* = 0.384) between coated and uncoated stents as well as between the different stent groups regarding endothelial cell loss, surface fibrin/platelet deposition, medial smooth muscle cell loss, or adventitial fibrosis were found. Acute self-limiting thrombus formed on 6/16 implants (37.5%), and all of the thrombi were noted on devices implanted in the common or external carotid artery irrespective of the surface coating. Two of 12 p64 HPC-coated stents (16.7%) and 1/4 uncoated p64 stents (25%) showed severe or complete stenosis at delayed angiography.

**Conclusions:**

In these preliminary *in vivo* experiments, HPC-coated p64 FDSs appeared to be biocompatible, without acute inflammation.

## Key points

- The hydrophilic coating (HPC) can be applied to p64 flow-diverter stents.
- The HPCs do not cause acute inflammation in the vessel wall.
- Hydrophilic coated p64 flow-diverter stents appear to be biocompatible in initial *in vivo* tests.

## Background

The introduction flow-diverter stents (FDSs) to the arena of interventional neuroradiology represented one of the most important breakthroughs for the specialty in recent times. These devices allowed not only the treatment of intracranial aneurysms but also the reconstruction of the parent vessel. Similarly, these devices are also being used in the peripheral circulation [1].

Although the exact mechanism of action of FDSs is unknown, it is believed they have a biphasic mechanism. Initially, the FDS redirects flow away from the aneurysm and promotes intra-aneurysmal stasis and thrombosis, which stabilises the aneurysm; subsequently, neointimal growth along the FDS struts remodels the vessel wall and completes the exclusion of the aneurysm from the circulation [2]. A wide variety of FDSs exists with newer devices entering the marketplace designed to target specific problems. One issue that is yet to be conclusively resolved is the need for antiplatelet medication when FDSs are implanted. This necessary medication is not without inherent risks. Similarly, there is hesitancy among the neuroradiological and neurosurgical community regarding antiplatelet medication in the presence of acute subarachnoid haemorrhage. Therefore, an optimised FDS would not require antiplatelet medication.

Various stent coatings have been extensively tested for stents used in the peripheral and cardiac circulation since the early 2000s [3–7] with pre-clinical studies published throughout the preceding decade [8–14]. Recently, the pipeline embolisation device Shield (Medtronic, Dublin, Ireland) has entered the market. This device, the first FDS with a thromboresistant coating, has a 3-nm-thick covalently bound phosphorylcholine surface modification. Phosphorylcholine is a major component of the outer membrane of erythrocytes and has demonstrated efficacy in resisting platelet adhesion as well as intimal hyperplasia [15–17]. Although there is limited clinical information available regarding the clinical results of this new technology [18–20], it is imperative to continue the development of antithrombogenic coatings that would minimise or completely negate the requirement for antiplatelet medications.

We have recently shown that the hydrophilic coatings (HPCs) have antithrombogenic properties when tested *in vitro* [21], but there is little known about the *in vivo* biocompatibility of these coatings. We sought to determine the acute therapeutic efficacy and biocompatibility of three different hydrophilic coatings. We present the results of *in vivo* testing of three different proprietary HPCs (type 1, 2, and 3). We assessed the biocompatibility of uncoated and coated p64 FDSs (Phenox, Bochum, Germany) without antiplatelet medication, single antiplatelet medication (acetylsalicylic acid (ASA)), and dual antiplatelet medication (DAPT) (ASA and clopidogrel).

## Methods

### Animal experiments and premedication

After Institutional Animal Care and Use Committee approval, four domestic male dogs, of similar age, were commenced on three different oral antiplatelet medication regimes. One dog did not receive antiplatelet medication, two dogs received only ASA 1.5 mg/kg/day, and one dog received DAPT (ASA 1.5 mg/kg/day and clopidogrel 1.5 mg/kg/day). The medication was commenced 72 h prior to the planned intervention and continued for the duration of the study. The canine model was chosen as the supra-aortic vessels have an appropriate diameter for clinically available stents and flow diverters. Furthermore, in common with humans, dogs lack spontaneous endothelialisation and have a variable-enhanced coagulability that would allow a suitable assessment of the different FDSs and antiplatelet regimes. The canine model has been used previously to investigate the treatment of aneurysms with FDSs [22–25].

### Flow-diverter implant procedure

All procedures were performed with the animals under general anaesthesia with acepromazine (0.2 mg/kg, intramuscularly), Telazol (5 mg/kg, intravenously), and maintenance with 2% isoflurane. The right common femoral artery was surgically exposed and a 6-Fr introducer sheath inserted. Using a 5-Fr vertebral catheter and standard 0.035-in. guidewire, angiography of the common carotid arteries (CCAs), external carotid arteries (ECAs), and subclavian arteries (SAs) was performed. After full heparinisation and activated clotting time 2–2.5 times the normal value, a 0.027-in. Marksman microcatheter (Medtronic, Dublin, Ireland) or Excelsior XT 27 (Stryker, Kalamazoo, USA) with 0.014-in. microwire was used to access the supra-aortic vessels.

### Flow-diverter stent characteristics

The p64 is a braided flow-diverting stent composed of 64 nickel-titanium (NiTi, nitinol) wires. Two platinum wires wrapped around the shaft assist in radio-opacity. The 64 wires are grouped into 8 bundles proximally, with each bundle consisting of 8 wires. A radio-opaque marker is attached to the end of each bundle. The porosity of the device is 51–60%.

### Surface HPCs

The initial results of *in vitro* testing of the HPCs were recently published [21]. In brief, it has been demonstrated that the coatings could be applied to both nitinol plates and nitinol wires that were subsequently used to construct p64 and p48 flow-diverter stents. The thickness of the surface coatings is from 10 to 20 nm as determined by x-ray photoelectron spectroscopy analysis. The thin nature of the coatings has no influence on the surface texture of the nitinol wires used to construct the flow-diverter stents [21]. *In vitro* testing showed a significant reduction in the adherence of immunofluorescent CD61-positive platelets when incubated with whole blood from healthy volunteers compared to uncoated stents. Scanning electron microscopy also demonstrated minimal adherent platelets on the coated flow diverters compared to a thick layer of adherent platelets on uncoated stents [21]. To summarise, the main differences among HPC-1, HPC-2, and HPC-3 are the following: HPC-1 is a well-known polyethylene glycol (PEG)-based coating, HPC-2 is a newly developed glycan-based multilayer polymer coating, and HPC-3 is a polyphosphazene nanocoating.

### Implant location

A total of 16 p64 flow-diverter stents were implanted in 4 animals with 4 stents implanted into each animal. In each animal, an uncoated p64 and 1 each of the HPC-1 p64, HPC-2 p64, and HPC-3 p64 FDSs were randomly assigned and implanted into segments of the ECA, CCA, or SA. Two implants were placed in the SAs, and 2 implants placed in the CCAs or ECAs. The devices were deployed under fluoroscopic guidance. The mean diameter of the CCA was 3.7 ± 0.23 mm (mean ± standard deviation, the ECA 3.52 ± 0.45 mm, and the SA 3.79 ± 0.36 mm). The implant locations and medications are summarised in Table 1. Control angiography was performed following implantation of the FDS and 45–60 min after the FDS implantation.

**Table 1:** Test matrix of the canine models presenting the medication regimens and the locations of each stent

Animal number	Medication	Right CCA/ECA	Left CCA /ECA	Right SA	Left SA
1	None	p64 HPC-1	p64 uncoated	p64 HPC-2	p64 HPC 3
2	ASA	p64 HPC-1	p64 uncoated	p64 HPC-2	p64 HPC 3
3	ASA	p64 HPC-2	p64 HPC-1	p64 HPC-3	p64 uncoated
4	ASA + clopidogrel	p64 HPC-2	p64 HPC-1	p64 HPC-3	p64 uncoated

*ASA* acetylsalicylic acid, *CCA* common carotid artery, *ECA* external carotid artery, *SA* subclavian artery

### Follow-up imaging

Sonographic imaging was performed to assess vessel patency on days 7, 14, and 24 after the procedure. Angiography was performed on day 28 after the procedure via the contralateral common femoral artery. Stents were graded as patent (no stenosis), with minimal stenosis (1–29% lumen diameter reduction), with moderate stenosis (30–49% lumen diameter reduction), with severe stenosis (50–99% lumen diameter reduction), and occluded (100% lumen diameter reduction). All angiographic studies were analysed by a single reader (AS) with over 15 years of experience in cerebral angiography.

### Harvest and gross imaging

Euthanasia was performed by intravenous sodium pentobarbital overdose (100 mg/kg) at 28 days whilst the animals were under anaesthesia with isoflurane and following the final angiographic images. The arterial segments with the implanted FDS were surgically removed and fixed in 10% formaldehyde. All specimens were photographed and radiographed using a LX-60 cabinet radiography system (Faxitron, Arizona, USA).

### Histopathology preparation

After gross imaging, the excised arterial segments were dehydrated in a graded series of ethanol and embedded in Spurr’s epoxy resin. After polymerisation, the transverse section from the proximal, middle, and distal ends of the FDS were taken and the cross sections adhered to plastic slides and prepared to a thickness of 32–88 μm (Exakt, Oklahoma City, USA). The slides were then polished and stained with haematoxylin and eosin stain.

### Histological and morphological assessment

Morphometric analysis was performed on each segment by an independent, experienced (> 15 years) histopathologist (RV) using digital planimetry with a calibrated camera. For each prepared section, a morphometric analysis was performed and included the luminal area of the vessel, the area of the internal and external elastic laminae (IEL and EEL, respectively), and the neointimal thickness that was calculated as the distance from the IEL to the luminal border. Semiquantitative data including surface platelet/fibrin deposition; injury; medial smooth muscle loss; neointimal, medial, and adventitial inflammatory cell invasion; and medial plus adventitial haemorrhage were recorded (Table 2). Histopathological analysis was conducted by an independent pathologist (RV) blinded to the coatings.

**Table 2:** Description of semiquantitative histology scores

	0 (none)	1 (minimal)	2 (mild)	3 (moderate)	4 (severe)
Endothelium					
Endothelial loss	None	< 25% of the circumference	25–50% of the circumference	51–75% of the circumference	> 75% of the circumference
Tissue matrix					
Surface (fibrin/platelet deposition)	None	Minimal, focal	Mild, multifocal	Moderate, regionally diffuse	Severe, marked diffuse, or total luminal occlusion
Inflammation					
Intima/media	None	< 20 inflammatory cells/HPF in < 25% of area	21–100 inflammatory cells/HPF in 25–50% of area	101–150 inflammatory cells/HPF > 51–75% of area	> 150 inflammatory cells/HPF > 75% of area
Adventitia	None	< 25% of area	25–50% of area	> 51–75% of area	> 75% of area
Haemorrhage					
Media	None	Focal, occasional	Multifocal and regional	Regionally diffuse	100% red blood cells
Adventitia	None	Focal, occasional	Multifocal and regional	Regionally diffuse	100% red blood cells
Medial cell loss					
Medial smooth muscle loss (depth)	None	< 25% of medial thickness	25–50% of medial thickness	51–75% of medial thickness	> 75% of medial thickness
Medial smooth muscle loss (circumference)	None	< 25% of circumference	25–50% of circumference	51–75% of circumference	> 75% of circumference
Adventitial fibrosis					
Adventitial fibrosis	None	< 25% of the area	25–50% of the area	51–75% of the area	> 75% of the area
Medial injury/rupture					
Medial injury	None	Partial disruption involving the medial wall (focal disruption of the internal elastic lamina)	Complete medial disruption with containment (intact external elastic lamina)	Complete disruption of the arterial wall involving the media	Complete disruption of the arterial wall involving the media

*HPF* high-power field

The slides were stained with haematoxylin and eosin, and all sections were examined by light microscopy. Inflammatory cells were counted per area score as follows: 0/none (no inflammatory cells), 1/minimal (< 20 inflammatory cells per high power field in < 25% of area), 2/mild (21–100 inflammatory cells per high power field in 25–50% of area, 3/moderate (101–150 inflammatory cells per high power field in 51–75% of area, and 4/severe (> 150 inflammatory cells per high power field in > 75% of area).

Additionally, the media area (EEL area minus IEL area), neointimal area (IEL minus luminal area), and percent luminal stenosis (1 minus [luminal area/IEL area] × 100) were also calculated.

Biocompatibility, for the purpose of this study, was defined as a non-significant difference in the degree of inflammation between the uncoated p64 stents and the HPC-coated p64 stents.

### Statistical analysis

For morphometric measurements, ANOVA was used for unpaired comparisons to calculate the significance of differences between the cumulative frequency distribution of coating groups. Semiquantitative (ordinal) data (including surface platelet/fibrin deposition, injury, medial smooth muscle cell loss, neointimal/medial and adventitial inflammation, medial and adventitial haemorrhage, and fibrin) were compared using the non-parametric Wilcoxon/Kruskal-Wallis (rank sums) test (Table 2). A *p* value lower than 0.05 was considered statistically significant.

## Results

A total of 16 devices were implanted in 4 animals. There was no morbidity or mortality during the intervention or after the procedure (mortality and overall morbidity 0%). None of the dogs demonstrated any new neurological symptoms during the follow-up period (neurological morbidity 0%).

### Intra-operative angiography results

#### No antiplatelet medication

Two p64 FDSs (one uncoated and one HPC-1) were implanted in the CCA/ECA of animals that did not receive any antiplatelet medication. Transient small thrombi were seen on both of these devices during the implantation procedure; however, in both cases, the thrombus did not progress to complete occlusion of the vessel on the angiography performed at the end of the procedure. There were no thrombi seen on the HPC-2 p64 and HPC-3 p64 implants in the SAs.

#### Single antiplatelet and dual antiplatelet medication

**Fig. 1:**
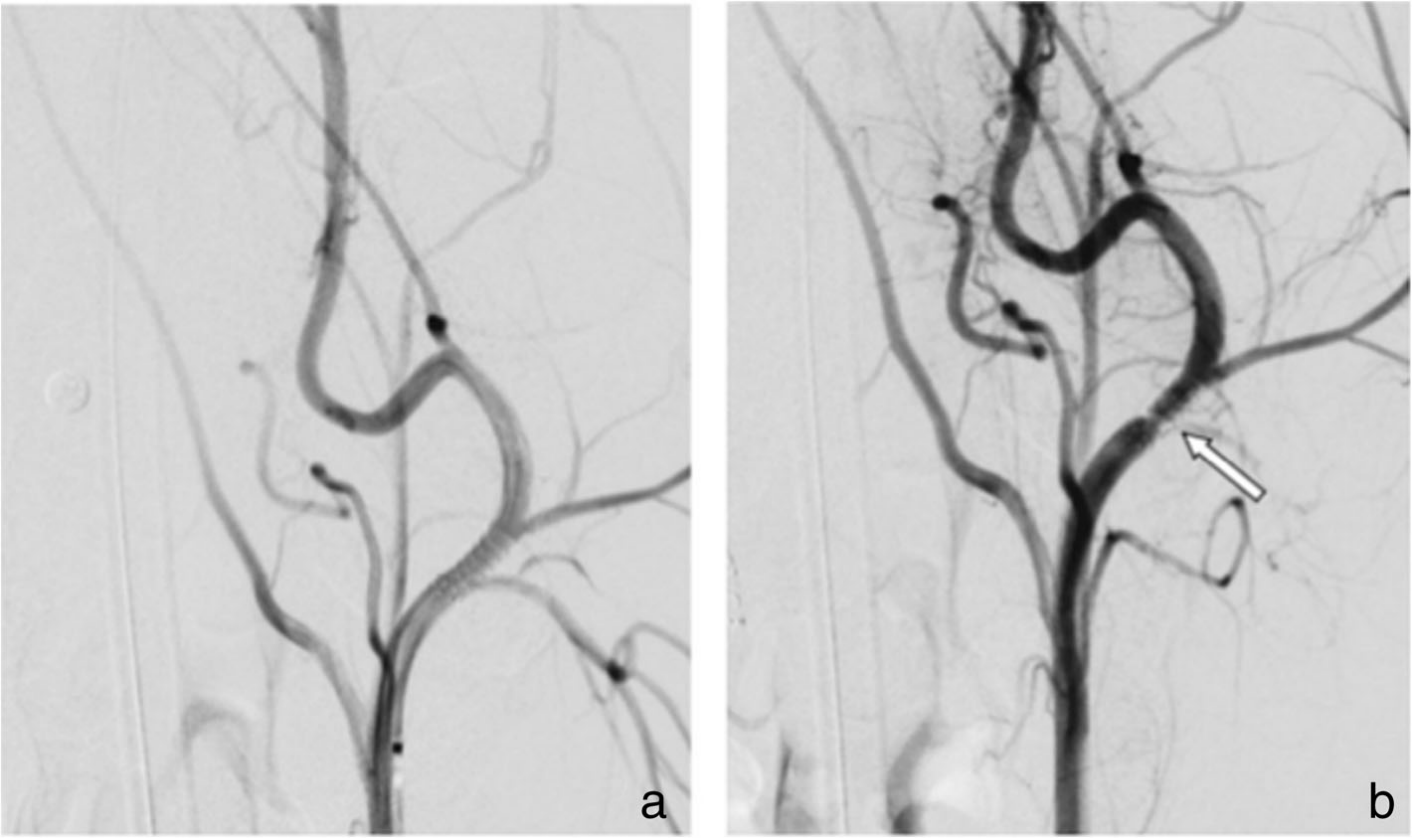
Subtracted angiographic series of the left external carotid artery of subject number 2 (treated with ASA only), implanted with an uncoated p64 device. **a** The FDS immediately after its implantation. **b** The formation of multiple thrombus in the acute phase (white arrow), 30 min after implantation

**Fig. 2:**
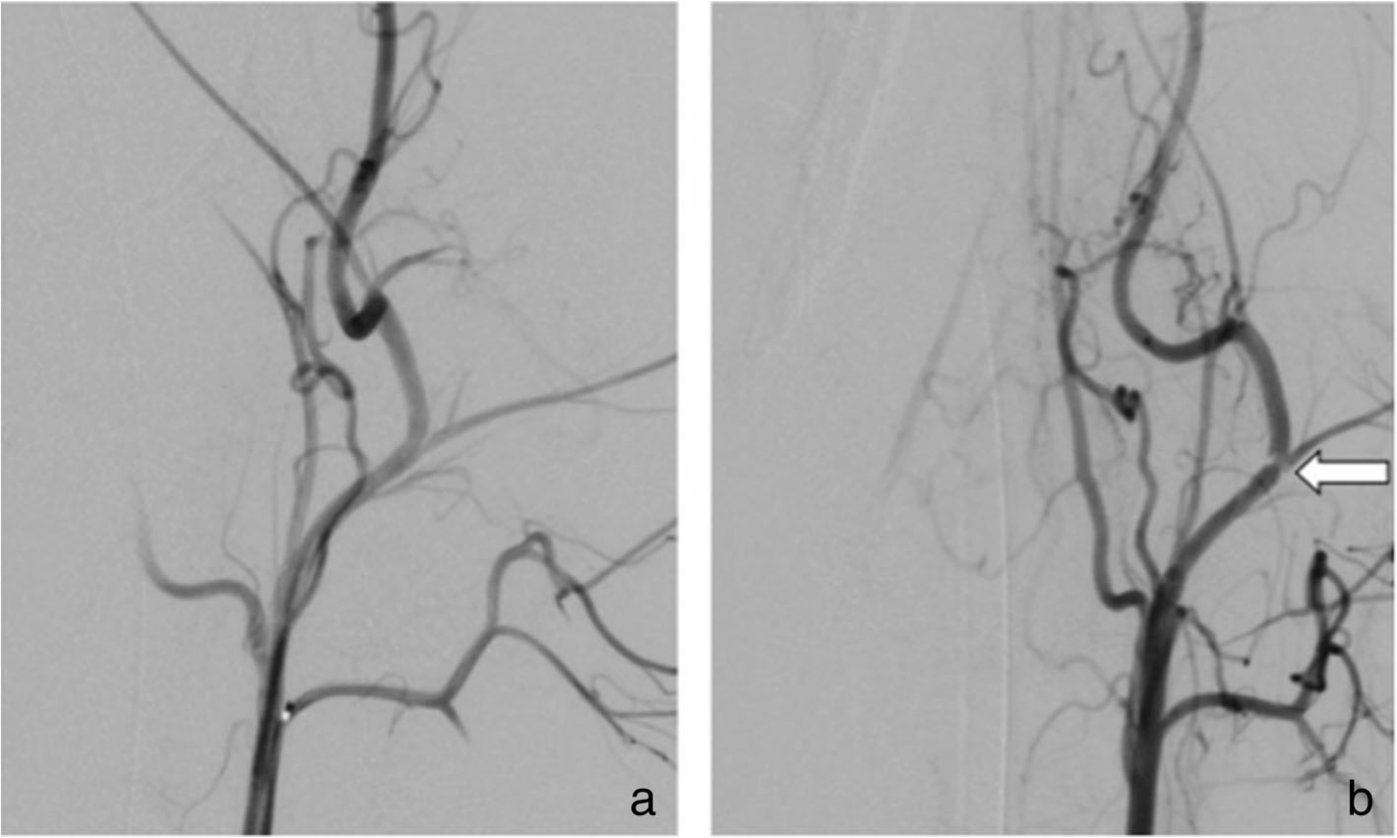
Subtracted angiographic series of the left external carotid artery of subject number 3 (treated with ASA only), implanted with an HPC-1 coated p64 device. **a** The FDS immediately after implantation. **b** The FDS 22 min after implantation, with a partial, acute in-stent thrombosis (white arrow)

**Fig. 3:**
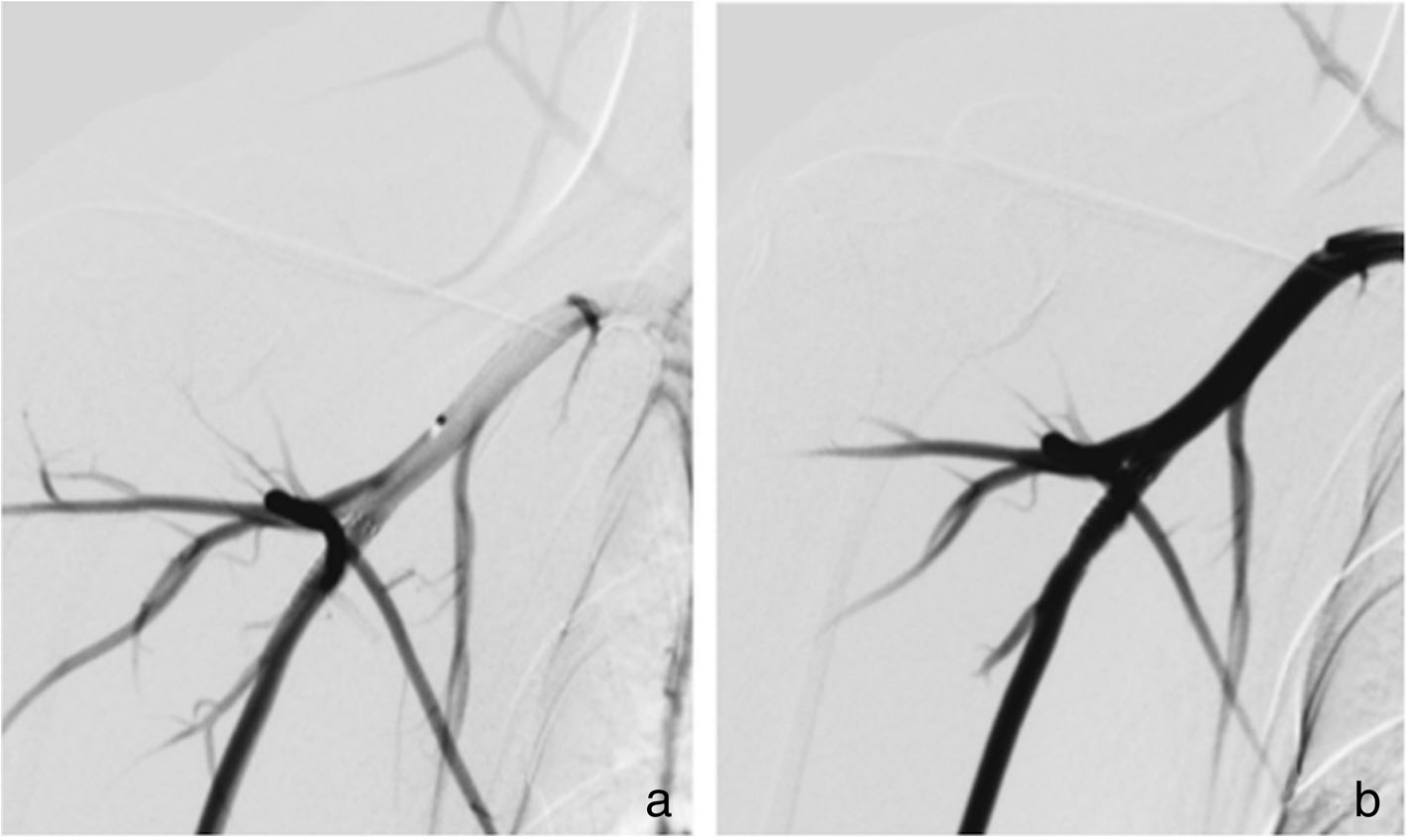
Subtracted angiographic series of the right subclavian artery of subject number 2 (treated with ASA only), implanted with an HPC-2 coated p64 device. **a** The FDS immediately after implantation, **b** 12 min thereafter. After the waiting test, the implant was free from thrombus

In the two animals given ASA only, four FDSs were implanted in the CCA/ECA (animal 2, HPC-1 p64 stent and uncoated p64 stent; animal 3, HPC-1 p64 stent and HPC-2 p64 stent). Transient small thrombi were seen on all the implants in the CCA/ECA. There were no thrombi seen on the FDSs implanted in the SAs (animal 2, HPC-2 p64 stent and HPC-3 p64 stent; animal 3, HPC-3 p64 stent and uncoated p64 stents) (Figs. 1, 2, 3). There was no evidence of thrombus formation on any of the FDSs implanted in the animal receiving DAPT.

Overall acute self-limiting thrombus was seen to form on 6/16 (37.5%) of the implants, and all of the thrombi were noted on the FDSs implanted in the CCA/ECA irrespective of the surface coating.

### Delayed angiography outcome

Angiography was performed in all animals on day 28 post-procedure.

#### No antiplatelet medication

A small thrombus was noted on the HPC-1 p64stents that had been implanted in the right CCA/ECA. There were no thrombi seen on the other FDSs.

#### Single antiplatelet and dual antiplatelet medication

**Fig. 4:**
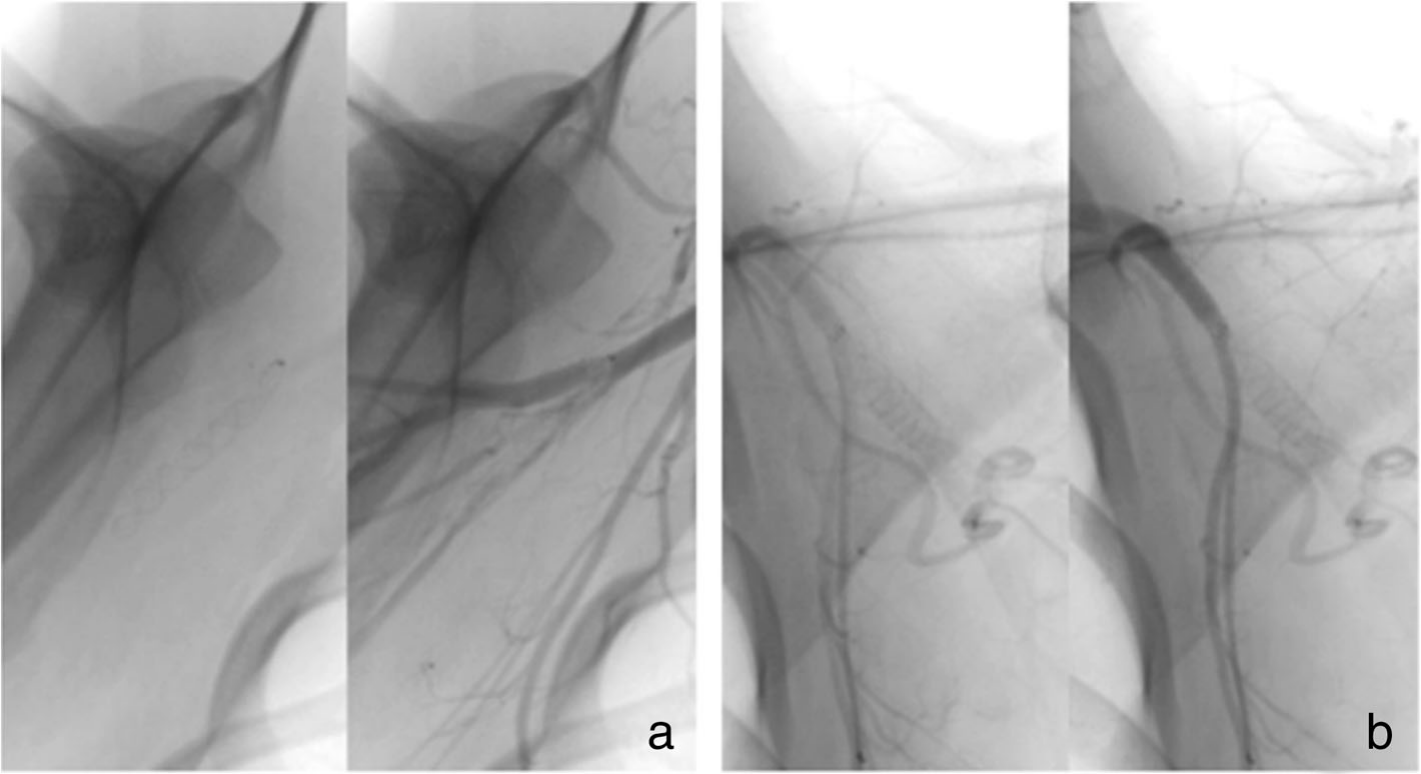
Unsubtracted angiographic series of the right subclavian artery (**a**) and left subclavian artery (**b**) of subject number 2 (treated with ASA only), implanted with HPC-2 and HPC-3 coated p64 devices, respectively, at day 28 (final angiographic control). **a** The right FDS almost occluded by an extensive in-stent thrombosis. **b** Complete occlusion of the left-sided device

**Fig. 5:**
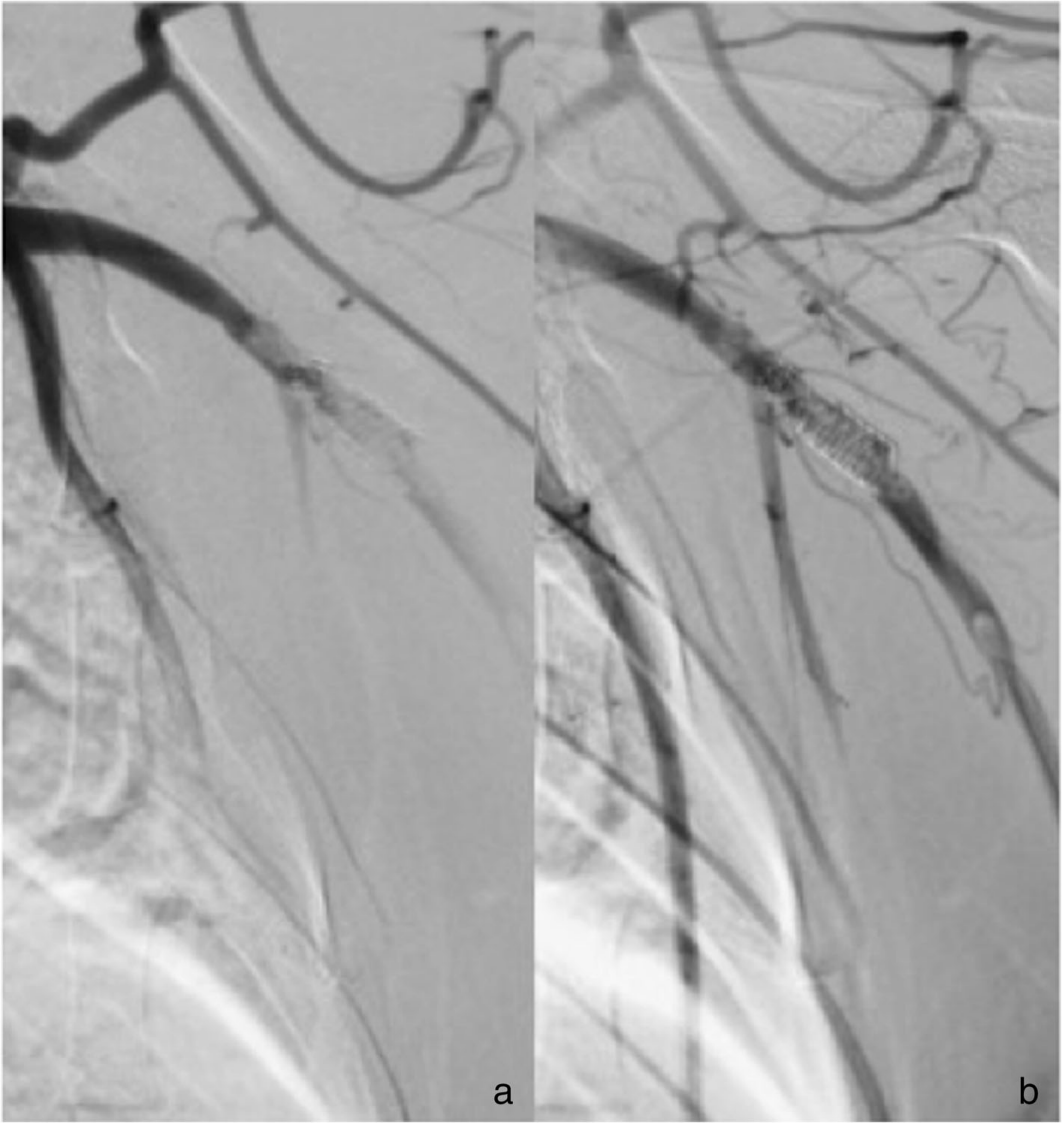
Unsubtracted angiographic series of the left subclavian artery of subject number 3 (treated with ASA only), implanted with an uncoated p64 stent, at day 28 (final angiographic control). It shows the occlusion of the stent lumen by moderate in-stent thrombosis (**a**) with subsequent impairment of the distal arterial flow (**b**)

In animal 2 (treated with ASA only), both the FDSs in the CCA/ECA were patent. The HPC-2 p64 stent in the right SA was completely occluded, and the HPC-3 p64 stent in the left SA was near-completely occluded on angiography. In animal 3 (treated with ASA only), the uncoated p64 stent in the left SA was near-completely occluded on angiography (Figs. 4 and 5). One device showed minimal (1–29%) stenosis, two devices showed severe (50–99%) stenosis, and one device showed complete stenosis on angiography performed at day 28. The cases of severe and complete stenosis were seen in animals treated with ASA only. There were no thrombi seen on any of the implanted FDSs in animal 4 (treated with DAPT).

The results of the end-procedural and delayed post-procedural angiography are summarised in Tables 3 and 4.

**Table 3:** Summary of the end-procedural angiography results

Animal number	Medication	Right CCA/ECA	Left CCA/ECA	Right SA	Left SA
1	None	p64 HPC-1 transient small thrombus	p64 uncoated, transient, small thrombus	p64 HPC-2, patent	p64 HPC-3, patent
2	ASA	p64 HPC-1, transient small thrombus	p64 uncoated, transient small thrombus	p64 HPC 2, patent	p64 HPC-3, patent
3	ASA	p64 HPC-2, small clot	p64 HPC-1, small clot	p64 HPC-3, patent	p64 uncoated, patent
4	ASA + clopidogrel	p64 HPC-2, patent	p64 HPC-1, patent	p64 HPC-, patent	p64 uncoated, patent

*ASA* acetylsalicylic acid, *CCA* common carotid artery, *ECA* external carotid artery, *SA* subclavian artery

**Table 4:** Summary of the delayed post-procedural (day 28) angiography results

Animal number	Medication	Right CCA/ECA	Left CCA/ECA	Right SA	Left SA
1	None	p64 HPC-1, minimal stenosis	p64 uncoated, patent	p64 HPC-2, patent	p64 HPC-3, patent
2	ASA	p64 HPC-1, patent	p64 uncoated, patent	p64 HPC-2, severe stenosis	p64 HPC-3, complete stenosis
3	ASA	p64 HPC-2, patent	p64 HPC-1, patent	p64 HPC-3, patent	p64 uncoated, severe stenosis
4	ASA + clopidogrel	p64 HPC-2, patent	p64 HPC-1, patent	p64 HPC-3, patent	p64 uncoated, patent

*ASA* acetylsalicylic acid, *CCA* common carotid artery, *ECA* external carotid artery, *SA* subclavian artery

### Gross and radiographic evaluation

**Fig. 6:**
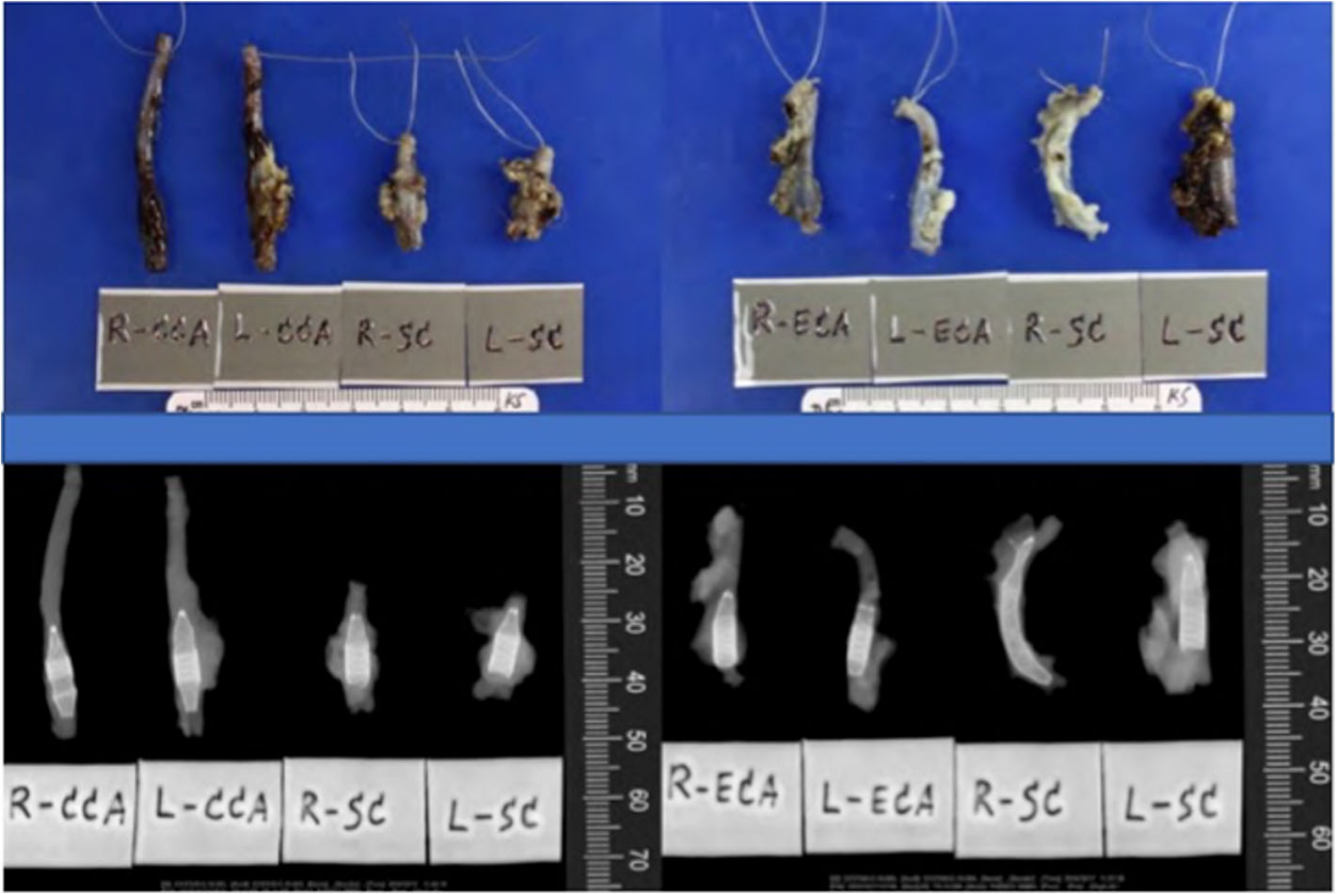
Macroscopic and radiographic analysis of the explanted vessel segments of subjects number 1 and 2

**Fig. 7:**
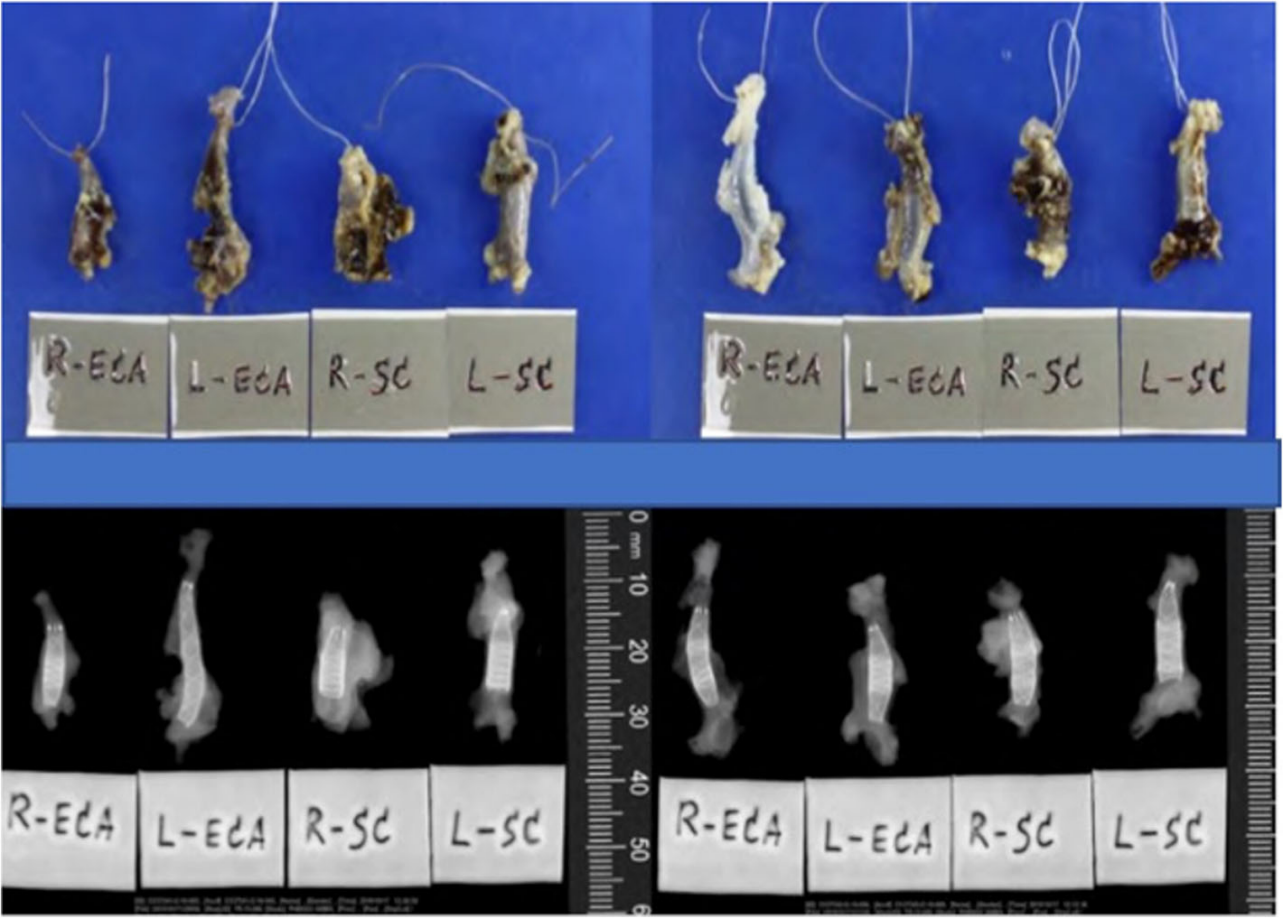
Macroscopic and radiographic analysis of the explanted vessel segments of subjects number 3 and 4

Mild dilatation of the arterial wall was noted correlating with the position of the implanted FDSs. There were no grossly visible perforations, lacerations, or erosions of the arterial wall although small areas of haemorrhagic discolouration were noted and thought to be due to the resection procedure (Figs. 6 and 7).

Radiography of the FDSs demonstrated them to be generally well opposed to the vessel wall. One of the proximal markers on the HPC-2 p64 FDS implanted in the right SA of animal 2 was deflected into a bifurcation branch (Fig. 6).

### Histopathological and morphological analysis

**Fig. 8:**
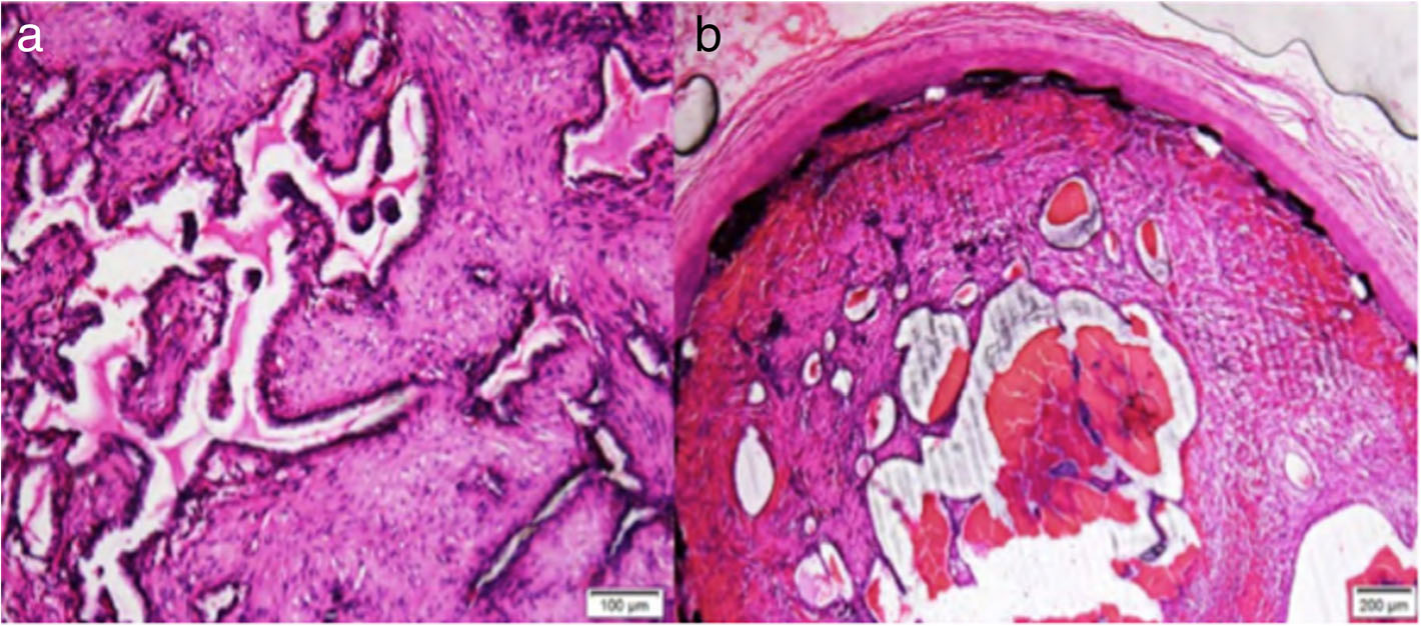
Sections from explanted HPC-2-coated device from subject number 2 show luminal occlusion with re-canalised organising (mid-segment) to organised (proximal and distal sections) thrombus (**a**). Chronic inflammatory cells infiltration around the mesh wires are visible (**b**). Haematoxylin and eosin stain, × 100 magnification

**Fig. 9:**
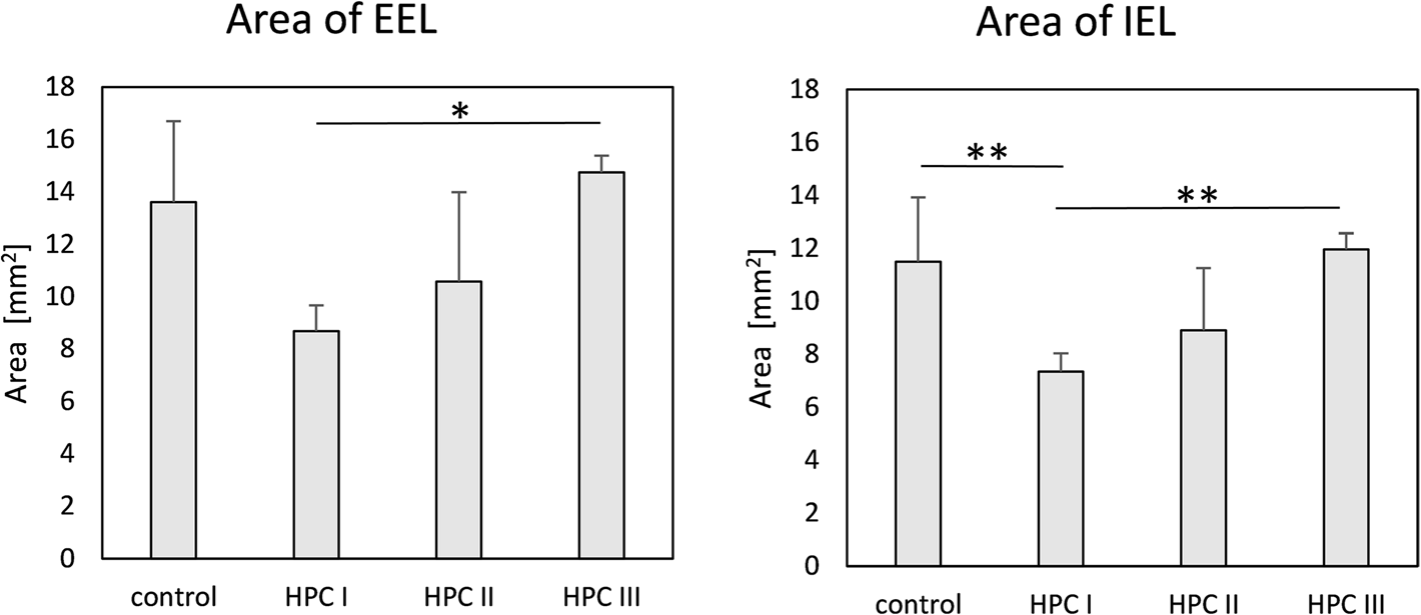
Histomorphometric measurements (mean ± standard deviations) of the external elastic lamina area (left) and the internal elastic lamina area (right). Asterisks denote statistical significance using ANOVA: *p < 0.05, **p < 0.01

All FDSs showed good apposition to the arterial wall and virtually complete neointimal coverage along the FDS strands. There was minimal inflammatory cell infiltration seen with no device-associated granulomatous cell inflammation (Fig. 8). Near-complete endothelialisation was noted for the patent FDSs (13/16 implants, 81.3%). Neointimal growth was noted in the patent FDS segments. Morphologically, there was a significant difference in the cross-sectional EEL area between the HPC-3 p64 and HPC-1 p64 (8.67 ± 0.99 mm^2^ versus 14.74 ± 0.64 mm^2^, *p* = 0.013) and the IEL area between the uncoated p64 and the HPC-3 p64 as compared to the HPC-1 p64 (11.96 ± 0.61 mm^2^, 11.49 ± 2.43 mm^2^ versus 7.34 ± 0.69 mm^2^, *p* = 0.009) (Fig. 9). The results are summarised in Table 5.

**Table 5:** Morphological measurements scoring

Coating group	EEL area (mm^2^)	IEL area (mm^2^)	Lumen area (mm^2^)	Neointimal area (mm^2^)	Medial area (mm^2^)	Stenosis (%)	Thickness (mm)
Uncoated (*n* = 4)	13.59 ± 3.10	11.49 ± 2.43	7.24 ± 4.98	4.24 ± 4.46	2.11 ± 0.78	36.40 ± 36.54	0.20 ± 0.17
HPC-1 (*n* = 4)	8.67 ± 0.99	7.34 ± 0.69	6.03 ± 0.64	1.30 ± 0.24	1.34 ± 0.50	18.58 ± 2.42	0.11 ± 0.03
HPC-2 (*n* = 4)	10.57 ± 3.40	8.89 ± 2.37	5.68 ± 4.09	3.22 ± 2.15	1.67 ± 1.29	41.39 ± 39.47	0.14 ± 0.06
HPC-3 (*n* = 4)	14.74 ± 0.64	11.9 6 ± 0.61	7.69 ± 4.00	4.27 ± 3.97	2.78 ± 0.21	37.06 ± 33.07	0.28 ± 0.27
*p* value	0.013 (HPC-1 versus HPC-3)	0.009 (HPC-1 versus HPC-3 + uncoated)	0.862	0.531	0.124	0.754	0.564

Data are mean ± standard deviation

*EEL* external elastic lamina, *IEL* internal elastic lamina

Histologically, there was no significant difference between the different stent groups regarding endothelial cell loss, surface fibrin/platelet deposition, medial smooth muscle cell loss, adventitial inflammation, or adventitial fibrosis. The results are summarised in Table 6.

**Table 6:** Histological scoring means, standard deviations, and Wilcoxon/Kruskal-Wallis results

Coating group	Endothelial cell loss	Surface fibrin	Neointimal/medial		Medial smooth muscle cell loss		Medial	Adventitial	
	Score	Platelets score	Fibrin score	Inflammation score	Depth	Circumference	Injury score	Inflammation score	Fibrosis score
Uncoated (*n* = 4)	0.08 ± 0.17	0.58 ± 1.17	0.83 ± 1.67	2.50 ± 1.04	0.25 ± 0.50	0.58 ± 1.17	0.42 ± 0.42	0.92 ± 1.26	0.00 ± 0.00
HPC-1 (*n* = 4)	0.17 ± 0.33	0.50 ± 0.58	0.00 ± 0.00	1.75 ± 0.74	0.00 ± 0.00	0.00 ± 0.00	0.25 ± 0.32	0.25 ± 0.32	1.50 ± 1.82
HPC-2 (*n* = 4)	0.50 ± 0.64	0.58 ± 0.69	0.33 ± 0.67	2.25 ± 0.57	0.50 ± 0.43	0.83 ± 1.23	0.50 ± 0.43	0.42 ± 0.63	1.50 ± 1.91
HPC-3 (*n* = 4)	0.25 ± 0.50	0.83 ± 1.26	0.92 ± 1.83	1.58 ± 0.96	0.42 ± 0.32	1.33 ± 1.22	0.58 ± 0.17	1.17 ± 0.88	0.00 ± 0.00
*p* value	0.731	0.963	0.762	0.384	0.183	0.144	0.514	0.522	0.181

## Discussion

The results of this initial *in vivo* study demonstrate no significant difference in the inflammatory response between the three different stent coatings compared to the uncoated p64 FDSs. There was no evidence of a severe inflammatory reaction or hypersensitivity reaction to either the HPC-1, HPC-2, or HPC-3 coatings which suggests that all three coatings have a similar biocompatibility to the uncoated nitinol p64 FDS. Similarly, the coatings did not elicit a fibrotic reaction within the adventitia. Although there was no significant difference in the endothelialisation between the different coatings, the HPC-1 p64 had the lowest neointimal area suggesting that endothelialisation on this coating may be impaired relative to the other stent coatings and the uncoated p64.

Kadirvel et al. [2] have previously shown that endothelialisation of implanted FDSs is important for reconstructing the parent vessel and ultimately excluding the treated aneurysm from the circulation. In this regard, a failure of endothelialisation could ultimately lead to a failure to occlude the treated aneurysms. This finding has previously been reported for fusiform aneurysms treated with FDSs [26], and inadequate neoendothelialisation has been suggested as the underlying cause for the failure of FDSs to complete occlude some aneurysms [27].

Therefore, the lack of endothelialisation seen on the surface of the HPC-1 p64 FDSs raises concerns regarding the potential use of this coating on FDSs. Conversely, the HPC-3 FDS had a similar neointimal area compared to the uncoated p64 FDSs (4.27 ± 3.97 mm^2^ versus 4.24 ± 4.46 mm^2^) which would suggest that neointimal growth on HPC-3 coated stents is similar to that on uncoated p64 stents.

In general terms, the initial event that leads to neointima formation is that of local thrombus formation, adjacent to the stent struts. Gradually, there is an invasion of cellular components such as macrophages and α-actin-negative spindle-shaped cells accompanied by the deposition of extracellular matrix components. This eventually differentiates into a fibrocellular lesion containing α-actin-positive smooth muscle cells. Therefore, mural thrombus with a subsequent macrophage infiltration after stenting may be crucial in recruiting smooth muscle cells from the arterial wall. Platelet adherence and aggregation promote the healing process through the release of growth factors and cytokines.

These observations strongly suggest that mural thrombosis with macrophage infiltration at the earliest stage after stenting may be crucial in recruiting smooth muscle cells from the arterial wall. Indeed, platelet adherence and aggregation promote the subsequent healing process through the release of growth factors [28–30]. Interestingly, the HPC-3 p64 FDS showed a non-significantly higher surface platelet/fibrin score, and it is possible that the HPC-3 coating allows enough platelet adhesion to promote initiation of the neointima formation but insufficient platelet adhesion to cause stent thrombosis. In the recent work of Matsuda et al., [31] the pipeline embolisation device Shield showed a greater stent coverage ratio, defined as the number of struts covered with neointima/total stent struts, compared to the pipeline embolisation device Flex (Medtronic, Dublin, Ireland) which is a similar device but without the phosphorylcholine coating. Similarly, the authors noted that the endothelial growth on the pipeline embolisation device Shield was faster than on the pipeline embolisation device Flex, and the authors suggest that this could be due to an effect of the coating. This is of interest since not only may this device reduce the risk of acute thrombogenic complications but it may also shorten the time that patients require antiplatelet medications. Although we have not conducted a similar experiment to date, it is possible that a similar effect could be seen with the HPC-3 coating and further experiments are ongoing.

We recognise that this study has limitations, and the translation of its conclusions into clinical practice needs to be carefully considered. The follow-up period of the study was just 1 month, and therefore, the medium- and long-term effect of the device coatings is not known. Similarly, FDSs are used to treat aneurysms, and in the current experiments, aneurysms were not created so any impact of the stent coating on aneurysm occlusion is unknown. Furthermore, a limited number of subjects were used, and larger cohorts would be necessary to prove the effect of the different coatings. The large number of variables and small sample size represent another major limitation of the study.

In conclusion, HPC surface-modified p64 FDSs are biocompatible in this animal study with no evidence of severe systemic allergic reaction, local inflammatory reaction, or significant fibrosis.

## Abbreviations

ASA: Acetylsalicylic acid
CCA: Common carotid artery
DAPT: Dual antiplatelet medication
ECA: External carotid artery
EEL: External elastic lamina
FDS: Flow-diverter stent
HPC: Hydrophilic coating
IEL: Internal elastic lamina
SA: Subclavian artery
